# A Novel Technique for Identification of Wear Values at Different Lengths after Multiple Clinical Use of Different File Systems

**DOI:** 10.3390/medicina58081117

**Published:** 2022-08-18

**Authors:** Neslihan Yılmaz Çırakoğlu, Ersan Çiçek, Cevat Özarpa, Yağız Özbay, Olcay Özdemir

**Affiliations:** 1 Department of Endodontics, Faculty of Dentistry, Karabük University, Karabük 78050, Turkey; 2 Private Dentist, Samsun 55070, Turkey; 3 Department of Mechanical Engineer, Karabük University, Karabük 78050, Turkey

**Keywords:** cone fitting, file deterioration, USB Micron Microscope, wear

## Abstract

*Introduction* The purpose of this research is to assess the wearing of the rotary file system (Protaper Next) and reciprocating file systems (Reciproc Blue and WaveOne Gold) at different lengths using a novel technique after in vivo clinical use. *Materials and Methods* Twelve different unused samples from each brand were accepted as reference values. For three different brands, the diameters of the files were measured by taking 12 samples used once, 12 samples used twice, and 12 samples used three times. Images were taken with a USB Micron Microscope, and file diameters were measured by determining limit values with Autocad. *Result* Reciproc Blue system was the most worn at apical 1 mm, and WaveOne Gold system was the most worn at apical 3 mm. PTN system exhibited the least wearing at any length. Moreover, less wearing was observed in the rotation motion than in the reciprocating motion. *Conclusion* In clinical practice, for the guttapercha to be fully adapted to the apical construction prepared according to the determined WL, the file should not undergo any wearing and volume reduction. Wearing—especially in the apical parts of the file—causes less preparation, and this situation could lead to apically obturation failure.

## 1. Introduction

Adaptation of nickel-titanium (Ni-Ti) metallurgy in the field of dentistry, especially for endodontic instruments, has provided critical developments [1]. The essential mechanical characteristics of this alloy are shape memory and superelasticity; however; to develop the mechanical characteristics of Ni-Ti systems, for instance, flexibility, resistance to cyclic fatigue and probability of fracture, different manufacturing procedures have been continuously introduced [2]. Furthermore, the alloy is composed of different thermomechanical treatings, alterations in the chemical and manufacturing ways of the alloy, and cross-sectional design. Therefore, M-wire alloy based on Ni-Ti was introduced, and it was reported that it provides better flexibility and fatigue resistance than those from traditional Ni-Ti systems [3]. On the market, there are various M-Wire Ni-Ti file systems with different brands or working kinematics (rotation or reciprocation) such as ProTaper Next (PTN, Dentsply, Maillefer, Ballaigues, Switzerland), Reciproc (VDW, Munich, Germany), and WaveOne (Dentsply Sirona, York, PA, USA) [4].

PTN files have progressive and regressive tapers and off-centered rectangular design. Variation in tapers minimizes the connection between the file and the dentin, which reduces inconvenient taper lock and the screw impression. Moreover, compared to a file with a centered mass and axis of rotation, the offset design of the PTN files maximizes the debris forced out of the canal. The PTN system comprises five files: X1 (tip size 17 with a taper of 0.04), X2 (tip size 25 with a taper of 0.06), X3 (tip size 30 with a taper of 0.07), X4 (tip size 40 with a taper of 0.06) and X5 (tip size 50 with a taper of 0.06) [5].

With the development of metallurgy technology, thermal processes have been applied to Ni-Ti systems for improving the mechanical properties of the alloy [6]. As a result, heat treatment has revealed Ni-Ti systems with different alloy structures and surface characteristics with different mechanical properties [7].

The Reciproc Blue system was manufactured from the Ni-Ti alloy by treating a few heating processes. The clear blue oxide stratum on the surface of the instrument was designed especially by heating and cooling procedures. Although the conception of the instrument is similar to that of the M-Wire Reciproc instrument, the Reciproc Blue system has more cyclic fatigue resistance and is more flexible than the M-Wire Reciproc system, according to previous studies [8,9].

WaveOne Gold (WOG, Dentsply/Tulsa Dental Specialties, Tulsa, OK, USA) systems that use the same reciprocating motion as WaveOne (Dentsply/Tulsa Dental Specialties) have been introduced. Their exclusive gold color results from a thermal cycling procedure (heated and cooled slowly many times). WaveOne Gold instruments have a parallelogram cross-sectional design, with 85 degrees in its cutting angles and 95 degrees in the non-cutting angles, including four instruments (20/0.07 (small), 25/0.07 (primary), 35/0.06 (medium) and 45/0.05 (large)) [10].

The manufacturing procedures of Ni-Ti instruments may affect the degree of fatigue and surface deterioration, leading to stress zones associated with crack initiation and propagation [11]. Generally, more flexible Ni-Ti files are less resistant to torsional loading [12]. Contrary to stainless steel, Ni-Ti instruments may fracture without visible defects; therefore, macroscopic inspection presents limited pre-evidence [13]. Anatomic configuration and the curvature degree of the root canal, besides instrument alloy, size, and taper, are critical in resistance to deformation and deterioration [14].

There is no research evaluating the amount of wear of file systems with different characteristics and operating modes by different numbers of re-use in vivo in the literature. Moreover, only deformation and qualitative wear of files have been detected with devices such as atomic force microscope (AFM) and scanning electron microscope (SEM) and after multiple uses in the studies carried out so far [15,16]. However, these methods cannot measure the quantitative aspect of wear and the diameter differences that occur. Thereby, the purpose of this clinical research is to determine the wearing and diameter difference of the rotary file system (ProTaper Next) and recently introduced reciprocating systems (Reciproc Blue and WaveOne Gold) after in vivo re-use using a novel technique. The null hypothesis of the research was that there would be no difference in wear values of reciprocating and rotary nickel-titanium files after multi-use.

## 2. Experiment Details

The study protocol was approved by the Ethics Committee of Non-Interventional Clinical Research Board with a reference number 2020/284. In this study, three different endodontic instruments, including ProTaper Next X2 (25/0.06), Reciproc Blue R25 (25/0.08), and WaveOne Gold Primary (25/0.07), were examined (*n* = 36/group). The samples were examined for any existing defects under a stereomicroscope at 20X (OLYMPUS S2 × 12, Tokyo, Japan) after removal from the packages. The samples with any deformation on the surface and structure were excluded. In all groups, samples were divided into three subgroups (*n* = 12/subgroup) according to the number of use for instrumentation: (i) files *used in only one molar tooth, (ii) files used in two molar teeth, and (iii) files used in three molar teeth.

The patients with root canals with an open apex, internal resorption or external resorption, severe dilaceration, and calcification and retreatment cases were excluded from the study. Moreover, teeth with root curvature not greater than 45 degrees and three roots and three canals were selected in order to provide standardization between different file systems.

After access cavities were prepared, the working length (WL) was determined with an electronic apex locator device (Root ZX mini; J Morita Co., Kyoto, Japan) and verified with a periapical radiograph. The patency was established using a K-file #10; glide path was performed with K-files #15 and #20 at the whole WL ProTaper Next files were used in the sequence of X1 (size 17/0.04) and X2 (size 25/0.06) with an endodontic motor (X–Smart Plus, Dentsply Maillefer, Ballaigues, Switzerland) according to the manufacturer’s instructions. Reciproc Blue files were used with the VDW.GOLD RECIPROC Motor (VDW, Munich, Germany) at the specific proposed setting “RECIPROC ALL” mode. WaveOne Gold files were used with the X-Smart Plus Motor at the “WAVEONE GOLD” mode. During preparation, until resistance was felt in the canal, a kind in-and-out brushing movement was used. After withdrawal from the canal, files were cleaned and checked visually before re-use. The same procedures were repeated until the file reached the WL. After three times pecking movement, the instruments were taken away, the debris was removed, and the root canals were irrigated with 3 mL 5.25% NaOCI solution during the instrumentation. One trained operator achieved all the root canal preparation. After each re-use, the samples were cleaned with disinfecting agent with an endodontic brush and kept in an ultrasonic bath (ODONTOBRA’S1) for 25 min with a heating system using an enzymatic detergent diluted in water to 5 mL per liter. After drying, the files were packed in a sterilization package marked for the subgroup and were sterilized at 134 °C under 30 psi for 20 min in an autoclave (Sterilix Vacuum Plus; Reverberi, Barco, Italy).

**Figure 1:**
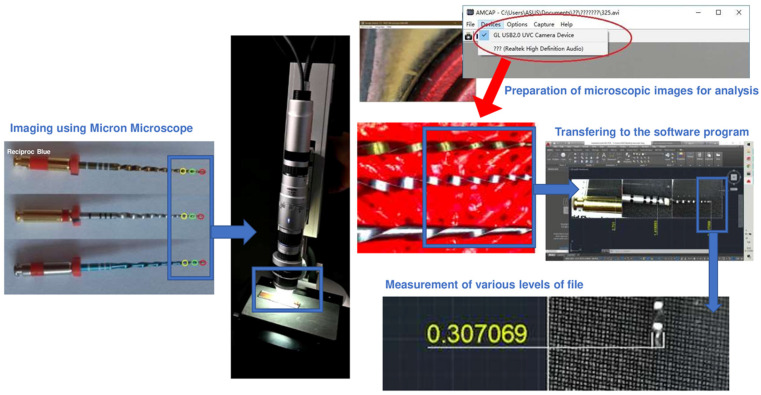
Stages of evaluation of wear of files caused by instrumentation.

USB Micron Microscope (HRX-01, Hirox, Japan) was used to measure the diameters of the files in the 1600× zoom with a holder prepared using a 3D printer (Zortrax M200 Plus, Olsztyn, Poland) for the standardized measurements. Moreover, AMCAP and Hvcap applications were used to capture microscope images. Measurements were taken between the limit values with the computer-aided design program Autocad (Autocad Corporation, 2018), which can measure by comparing the images taken from the applications. The wear of file, measurements, and calculations were made at 1 mm, 3 mm, and 5 mm from the apical portion. The areas marked in Figure 1 indicate the following: red circle—the first millimeter; green circle—the third millimeter; yellow circle—the fifth millimeter from the apical measurements were taken. Thirty-six samples from three different file systems, PTN, Reciproc Blue, and WaveOne Gold, were measured for a total of 108 samples. Twelve different unused files from each group were considered as references. In this study, 432 measurements were made on 144 files in total.

## 3. Statistical Analysis

Statistical analysis of the data was performed using MiniTab 17 Statistical Software (Statistical Software Release, version 17.3.1, Minitab Inc., State College, PA, USA). Descriptive statistics were obtained. In binary comparisons, normally distributed data were analyzed with *t*-test, and not normally distributed data were analyzed with Mann–Whitney U test. *p* values < 0.05 were considered to indicate statistical significance for all tests. The data were reported as mean ± standard deviation (mean ± SD) values (Table 1, Table 2 and Table 3).

## 4. Results

In all three file systems, there was a reduction in circumferential diameters at apical 1st, 3rd, and 5th millimeter in all files.

### 4.1. In the PTN File Group

At apical 1 mm, there was a significant decrease after the 1st, 2nd, and 3rd uses * *(p* < 0.05).

At apical 3 mm, there was a significant decrease between the 1st and 3rd uses in terms of diameter measurements * (*p* = 0.023).

### 4.2. In the Reciproc Blue Group

At apical 1 mm, there was a significant decrease after the 1st, 2nd, and 3rd uses * (*p* < 0.05).

At apical 3 mm, there was a significant decrease between the 1st and 3rd uses in terms of diameter measurements * (*p* = 0.003).

### 4.3. In the WaveOne Gold Group

At apical 1 mm, there was a significant decrease after the 1st, 2nd, and 3rd uses * *(p <* 0.05).

### 4.4. Comparing the Diameter Differences between the File Groups

In the 1st use of files, wearing at apical 1 mm was higher in Reciproc, WaveOne, and PTN groups, respectively. However, statistically significant difference was found only between PTN and WaveOne Gold groups * (*p* = 0.001).

In the 2nd use of files, wearing at apical 1 mm was higher in Reciproc, WaveOne, and PTN groups, respectively. In addition, a statistically significant difference was found between PTN and Reciproc Blue * (*p* = 0.026), PTN and WaveOne Gold groups * (*p* = 0.023).

In the 3rd use of files, wearing at apical 1 mm was statistically higher in Reciproc, WaveOne, and PTN groups, respectively. The *p* values of all results are shown in Table 1.

In the 1st use of files, wearing at apical 3 mm was higher in WaveOne, PTN, and Reciproc Blue groups, respectively. The WaveOne Gold group wore significantly higher than the Reciproc Blue group * (*p* = 0.0367) and the PTN group * (*p* = 0.049).

In the 2nd use of files, wearing at apical 3 mm was higher in WaveOne, PTN, and Reciproc Blue groups, respectively. The WaveOne Gold group wore significantly higher than the Reciproc Blue group * (*p* = 0.023) and the PTN group * (*p* = 0.034).

In the 3rd use of files, wearing at apical 3 mm was statistically higher in, WaveOne, PTN, and Reciproc Blue groups, respectively. 

**Figure 2:**
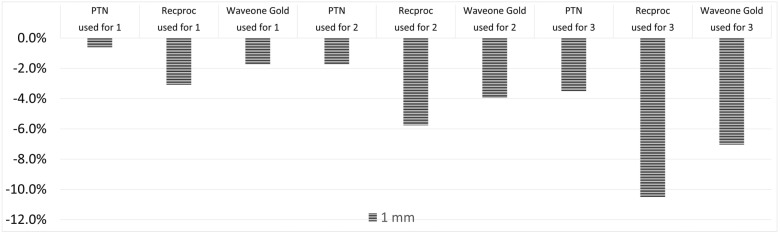
Comparison of the average wear values of the files were measured at 1st millimeter from the apical compared to the file that was never used between different file systems.

**Figure 3:**
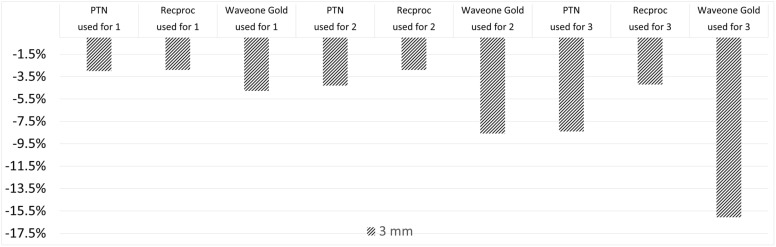
Comparison of the average wear values of the files were measured at 3rd millimeter from the apical compared to the file that was never used between different file systems.

**Figure 4:**
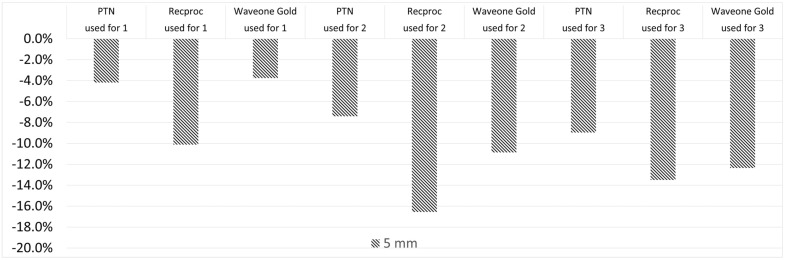
Comparison of the average wear values of the files were measured at 5th millimeter from the apical compared to the file that was never used between different file systems.

The 1st, 2nd, and 3rd uses of files wearing at apical 5 mm were higher in Reciproc, WaveOne, and PTN groups, respectively. However, there existed no statistically significant difference between file groups. Comparisons of the average wear values of the files were measured at 1st, at 3rd, and at 5th millimeters from the apical compared to the file that was never used between different file systems, as demonstrated in Figure 2, Figure 3 and Figure 4, respectively.

## 5. Discussion

Insufficient obturation of the root canal space may lead to a poor prognosis of the root canal treatment. Therefore, one of the most important factors of a successful prognosis of endodontic treatment is the ability to perform a hermetic root canal obturation [17]. To perform a hermetic seal of the root canal, root canal filling material, such as gutta-percha with root canal sealer, should obturate the root canal space. The practitioner verifies the gutta-percha cone suitability to the canal by feeling the ‘‘tug-back’’ sensation after the prepared root canal is ready to obturate [18]. Gutta-percha that is used for master apical gag may not be suitable for the canal chamber as a result of repetitive shaping processes and insufficient cutting efficiency. Therefore, the file should not undergo any wearing and volume reduction for the gutta-percha to fully adapt to the apical construction prepared according to the determined WL. Wearing—especially in the apical part of the file—causes less preparation than necessary. This situation may lead to tug back failure. 

The elements affecting surface wearing of file systems may be abstracted into the following factors: the number of use for instrumentation, the working principle, cross-sectional design, and the configuration of root canals in which the files were used [18]. Cutting capacity and the amount of wearing depend on the characteristics of each file system. Moreover, despite there being the same instrument, the cutting capacity and wearing may alter relevant to the number of file usage. Park et al. and Caballero et al. stated that the Reciproc files could be used in five or nine canals, respectively, without leading to structural defects [19,20]. These findings should be assessed with caution as mechanical defects of the file may decrease its biomechanical efficiency.

In this study, not only the amount of wearing of two different reciprocating and one rotary instrument system were compared, but the alteration in the effectiveness of the files and wearing according to the number of file usage was also compared. Findings indicated that in all three file systems, at the apical 1st millimeter level, the statistically significant wearing and diameter reductions occurred between the first, second, and third uses. At the apical 3 mm, a significant difference in wearing is observed between the 1st use and the 3rd use in the PTN and Reciproc systems; however, there was no difference observed in the WaveOne Gold group. At the apical 5 mm, there was no significant difference in terms of wearing between the 1st, 2nd, and 3rd uses in all three file systems.

PTN files present a snake-like curl action called “off-centered”. Moreover, they have a rectangular cross-section that allows them to be positioned in the center, and the manufacturer claims that PTN files are more flexible and more resistant to wearing [5]. In addition, it has a lower taper compared to WaveOne Gold and Reciproc Blue systems. These design characteristics may be claimed as the reason for the low amount of wear compared WaveOne Gold and Reciproc Blue systems.

The working kinematics of the file system may also play an important role in the effectiveness and wearing of the instrument [20]. With the reciprocating motion during the instrumentation, only one file may be able to perform all root canal preparation and increase of wearing when one might have to use so many sequences of files. Hence, the PTN system exhibiting less wearing occurred when compared to the file system that worked by reciprocating motion (Reciproc and WaveOne Gold).

The configuration of the root canal in which the instrument was used is one of the most clinically changeable situations. So, some researchers tested cutting efficiency in standardized resin blocks, not in actual teeth [21,22]. However, several recent studies reported a difference between cutting a resin block and the natural tooth structure [23,24,25]. Therefore, in this study for more clinical assumptions, the natural teeth that function in the mouth were included.

In previous studies, dental operation microscope, stereomicroscope, microcomputed tomography, AFM, SEM and mathematical finite element model were used to assess surface deformations in nickel-titanium alloys [11,26,27,28,29]. But in these methods, only deformation and qualitative wear were detected in the files after multiple-use ex-vivo conditions. These methods cannot measure the quantitative aspect of wear and the diameter differences in a 3D manner that occurs in clinical conditions. For these reasons, we used a USB micron microscope in this study with 1600× zoom. A USB microscope is an optical device equipped with a visual-to-digital converter. It enables the storage of a micro-object, including instantaneously transferring an image to a computer, recording a digital video, displaying it on the screen, printing it, and including it in a presentation. The USB Micron Microscope used for this study is used in clinical areas for agricultural units, from biological studies to monitoring chemical reactions. It can be used directly on insulating, non-insulating and living cell samples [30]. This USB Micron Microscope has a growth rate of 1600×. It can be zoom in with the buttons on it. The images looked at for the study can be followed from the computer with a USB connection. It is possible to use it on the computer by downloading the necessary software to monitor the images from the computer, such as hvcap, amcap. The microscope has its own lighting lamps. The light level can be adjusted with the adjustment keys on the microscope. It can be used for measurements used on a 1-micrometer scale. It provides 2D image resolutions down to 1 micrometer. It has a monocular eye head that can rotate 360 degrees. There is an adjustable lever on the microscope lenses to keep them in a fixed position. The adjustable lever keeps the system stable, making it easier to focus the microscope. It is possible to shoot with high resolution 1920 × 1080 scale image quality. It can be video recorded to facilitate later examination of images or enable the analysis of moving objects. The frame rate of the microscope is 30 per second. Video recording can be watched live on the computer [31].

We used reciprocating and rotary Ni-Ti instruments of the same apical size. The instrument of size #25 was chosen for apical shaping as well as for the final shaping, as this size is recommended for apical instrumentation in most nickel-titanium rotary systems.

There is limited data on the amount of wearing Waveone Gold and Reciproc Blue systems in the literature. According to the studies performed with file systems similar to the systems we examined in our research, AlRahabi and Atta observed the differences in the surface properties of WaveOne, WaveOne Gold, Reciproc, and Reciproc Blue file systems after root canal preparation [32]. They reported that WaveOne and WaveOne Gold instruments had significantly higher levels of surface defects after root canal instrumentation, and Reciproc and Reciproc blue had the lowest level of surface defects. Consistent with our study, the WaveOne Gold group wore significantly higher than the Reciproc Blue group at apical 3 mm level. Similar to the results of our study, Zafar reported that the post instrumentation evaluation showed significant differences in wearing values between the three groups (WaveOne Gold, PTN, and PTG), with WOG exhibiting higher values than PTN [33].

On the other hand, Van Pham and Vo reported that the surface roughness values of the WaveOne Gold Primary and Reciproc Blue R25 were not statistically changed after the first utilization in glide path created resin root canals [34]. The reason for this difference may be shown by the use of resin root canals instead of in vivo clinical natural teeth and by the use of surface roughness values (surface defects) as the wearing parameter instead of surface diameter measurements.

In conformity with the data from this study, Barbosa et al. reported that more defects located 3 mm from the instrument tips were identified in WaveOne primary instruments compared to Reciproc R25 [35]. Hanan et al. observed defects and deformations at 2 and 4 mm from the tips of Reciproc and Wave One instruments similar to our study [36]. However, in contrast to the results of our study, they reported that WaveOne files showed more defects and deformations than the Reciproc files after preparation in the two investigated lengths (0–2 and 2–4 mm). The reason for the conflicting results may be explained by using extracted teeth instead of in vivo clinical natural teeth. In vitro studies use more standardized structures of the samples by measuring and adapting. The main limitation of this study was the curvature, and calcification degrees of the root canals under clinical conditions could not be standardized. Moreover, the metallurgical and physical properties of the files used in our study (Reciproc Blue and WaveOne Gold) had different structures developed with up-to-date heat treatment technology compared to the files in that study.

Preclinical studies reveal preliminary results in an experimental standardized setup; however, they are insufficient to mimic the clinical effects. The fact that the files were evaluated after clinical use due to the design of this study may be considered as a limitation in terms of standardization. However, it is inevitable to arrange all parameters for both clinically in vivo and in vitro studies in the field of dentistry. Although this study provided limited standardization, it reflected all clinical conditions that make the results clinically relevant.

## 6. Conclusions

* The results of this study showed the wear amount of the different file systems after clinical use. Reciproc Blue system was the most worn at apical 1 mm, and WaveOne Gold system was the most worn at apical 3 mm. The PTN system exhibited the least wearing at any length. Moreover, less wearing was observed in the rotation motion than in the reciprocating motion.

* USB micron microscope analysis results showed that wearing—especially in the apical parts of the file—causes less preparation, and this situation could lead to apical obturation failure.

## Figures and Tables

**Table 1:** Measurements for at 1 mm from apical (mean ± SD).

Usage/Groups	PTN	WOG	RCB
First	0.00628 ± 0.00249 ^a,A^	0.017600 ± 0.001996 ^d,B^	0.03664 ± 0.02109 ^a,B^
Second	0.01756 ± 0.01183 ^b,C^	0.03928 ± 0.00701 ^e,D^	0.0576 ± 0.0288 ^g,D^
Third	0.03518 ± 0.01435 ^c,E^	0.07028 ± 0.00782 ^f,F^	0.10530 ± 0.01692 ^h,G^

Different characters indicate statistically significant difference (*p* < 0.05).

**Table 2:** Measurements for at 3 mm from apical (mean ± SD).

Usage/Groups	PTN	WOG	RCB
First	0.02998 ± 0.01770 ^a,A^	0.04776 ± 0.01959 ^c,B^	0.01516 ± 0.00359 ^f,A^
Second	0.04302 ± 0.02049 ^a,b,C^	0.0859 ± 0.0539 ^d,D^	0.0249 ± 0.0283 ^f,g,C^
Third	0.0953 ± 0.043 ^b,E^	0.1354 ± 0.0850 ^e,E^	0.03008 ± 0.00511 ^g,F^

Different characters indicate statistically significant difference (*p* < 0.05).

**Table 3:** Measurements for at 5 mm from apical (mean ± SD).

Usage/Groups	PTN	WOG	RCB
First	0.0419 ± 0.0580	0.03750 ± 0.01948	0.1011 ± 0.0680
Second	0.0740 ± 0.0710	0.1143 ± 0.0751	0.1168 ± 0.1022
Third	0.0897 ± 0.0589	0.1236 ± 0.1034	0.1349 ± 0.0989

There existed no statistically significant difference between file groups (*p* > 0.05).

## Data Availability

Not applicable.
